# Androgen Receptors in Epithelial Cells Regulate Thymopoiesis and Recent Thymic Emigrants in Male Mice

**DOI:** 10.3389/fimmu.2020.01342

**Published:** 2020-06-29

**Authors:** Anna S. Wilhelmson, Marta Lantero Rodriguez, Inger Johansson, Elin Svedlund Eriksson, Alexandra Stubelius, Susanne Lindgren, Johan Bourghardt Fagman, Pamela J. Fink, Hans Carlsten, Olov Ekwall, Åsa Tivesten

**Affiliations:** ^1^Wallenberg Laboratory for Cardiovascular and Metabolic Research, Department of Molecular and Clinical Medicine, Institute of Medicine, University of Gothenburg, Gothenburg, Sweden; ^2^Center for Bone and Arthritis Research (CBAR), Institute of Medicine, University of Gothenburg, Gothenburg, Sweden; ^3^Department of Rheumatology and Inflammation Research, Institute of Medicine, University of Gothenburg, Gothenburg, Sweden; ^4^Department of Pediatrics, Institute of Clinical Sciences, University of Gothenburg, Gothenburg, Sweden; ^5^Department of Immunology, University of Washington, Seattle, WA, United States

**Keywords:** androgens, T cells, thymus, thymic epithelial cells, mice

## Abstract

Androgens have profound effects on T cell homeostasis, including regulation of thymic T lymphopoiesis (thymopoiesis) and production of recent thymic emigrants (RTEs), i. e., immature T cells that derive from the thymus and continue their maturation to mature naïve T cells in secondary lymphoid organs. Here we investigated the androgen target cell for effects on thymopoiesis and RTEs in spleen and lymph nodes. Male mice with a general androgen receptor knockout (G-ARKO), T cell-specific (T-ARKO), or epithelial cell-specific (E-ARKO) knockout were examined. G-ARKO mice showed increased thymus weight and increased numbers of thymic T cell progenitors. These effects were not T cell-intrinsic, since T-ARKO mice displayed unaltered thymus weight and thymopoiesis. In line with a role for thymic epithelial cells (TECs), E-ARKO mice showed increased thymus weight and numbers of thymic T cell progenitors. Further, E-ARKO mice had more CD4^+^ and CD8^+^ T cells in spleen and an increased frequency of RTEs among T cells in spleen and lymph nodes. Depletion of the androgen receptor in epithelial cells was also associated with a small shift in the relative number of cortical (reduced) and medullary (increased) TECs and increased CCL25 staining in the thymic medulla, similar to previous observations in castrated mice. In conclusion, we demonstrate that the thymic epithelium is a target compartment for androgen-mediated regulation of thymopoiesis and consequently the generation of RTEs.

## Introduction

Androgens, such as testosterone, are important modulators of the immune system and immune-related disorders (1). Androgens also suppress the number of peripheral T cells in both mice and men (2–5). Testosterone replacement lowers circulating T cells in hypogonadal men to levels equivalent to those of healthy controls (5) and in patients with Klinefelter syndrome, elevated T cell levels were normalized after testosterone supplementation (4).

T cell progenitors are produced in the bone marrow and then enrolled in thymopoiesis, i.e., further proliferation, selection, and maturation of T cells in the thymus. It is well recognized that androgens have a crucial impact on thymus size and contribute to the involution of the thymus taking place during puberty in both mice and humans. In androgen deficient states, both thymus size and thymopoiesis are prominently increased and the thymus involutes upon treatment with androgens (2, 3, 6–12). Recent thymic emigrants (RTEs), i.e., immature T cells that derive from the thymus and continue their maturation to mature naïve T cells in secondary lymphoid organs, are also regulated by androgens; the fraction of RTEs increases in the periphery after castration and/or androgen deprivation therapy of both mice and humans (3, 5). Besides being progenitors to mature T cells, RTEs have distinct properties and may play specific roles in immune disorders (13).

Despite the important actions of androgens on T cell homeostasis, understanding about relevant target cells remains incomplete. In a series of bone marrow transplantation experiments using the Tfm/Y mouse model, chimeric mice lacking a functional androgen receptor (AR) in non-hematopoietic cells showed increased thymus size, while mice lacking a functional AR in bone marrow-derived cells did not (6). It is therefore likely that AR in stromal cells and not in the hematopoietic compartment is important for the AR-mediated effects of androgens on the thymus. This notion has been strengthened by findings that a cell-specific knockout of AR in epithelial cells regulates thymopoiesis (14). Further supporting thymic epithelial cells (TECs) as an important androgen target, recent data suggest that castration of male mice alters the relative numbers of cortical TECs (cTECs) and medullary TECs (mTECs) (15). Given the central role of TECs for many thymic processes (16), we hypothesized that TECs are target cells for androgen-mediated regulation of both thymopoiesis and peripheral RTEs.

In this study, we have utilized the AR knockout (ARKO) mouse model to investigate how the AR mediates the effects of androgens on thymopoiesis and the peripheral T cell pool, using male mice with general- (G-ARKO) as well as T cell-specific (T-ARKO), and epithelial cell-specific (E-ARKO) knockout of the AR. Specifically, we asked the question whether AR in epithelial cells regulate RTEs in secondary lymphoid organs.

## Results

### Increased Thymopoiesis in G-ARKO Mice

**Figure 1:**
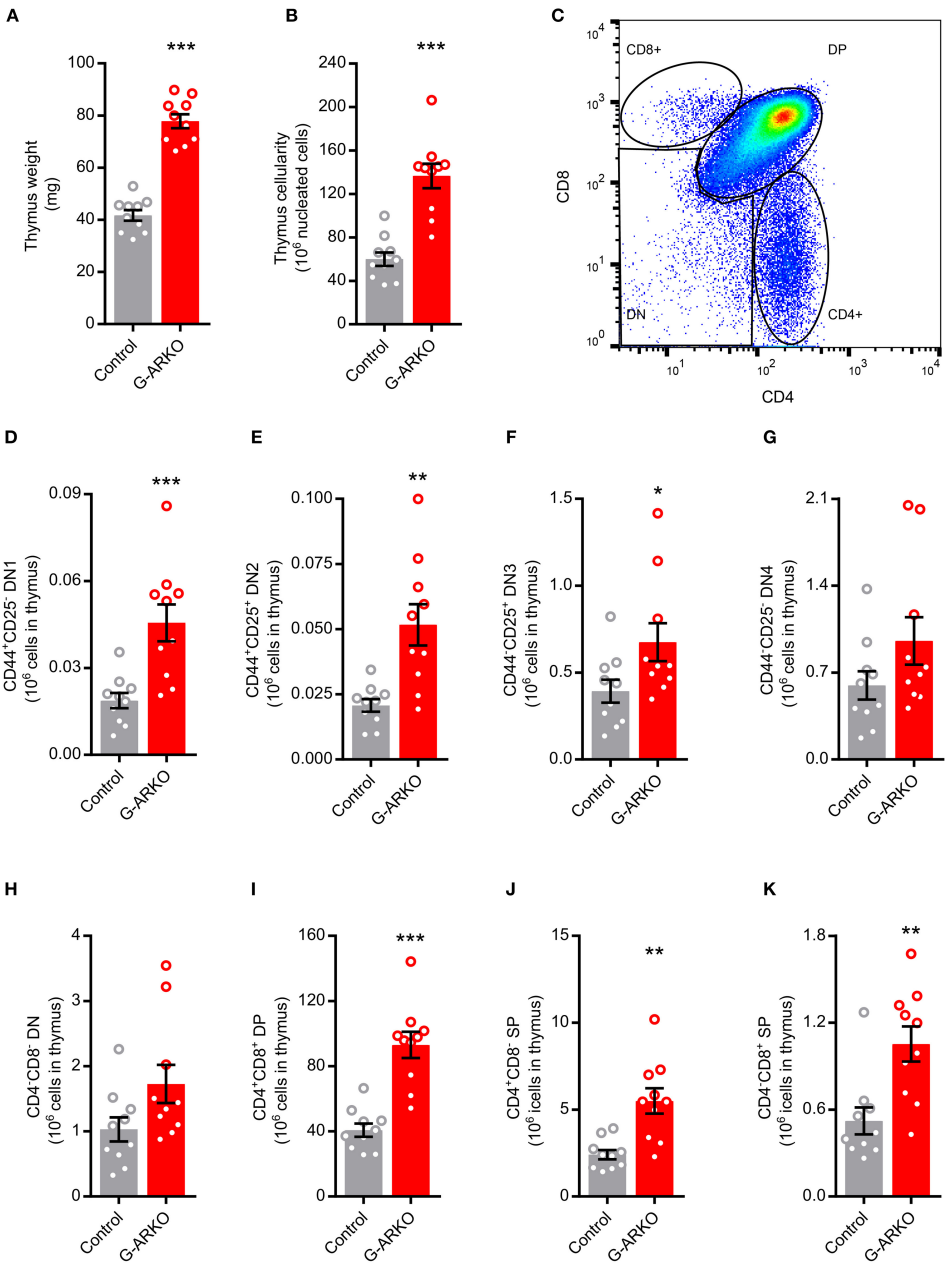
Increased thymopoiesis in mice with general depletion of the AR (G-ARKO). **(A,B)** Thymus weight and cellularity in control (Pgk-Cre^+^; *n* = 10) and general androgen receptor knockout (G-ARKO; AR^fl^Pgk-Cre^+^; *n* = 10) male mice. **(C)** Gating strategy for thymic subsets. **(D–G)** Number of CD4^−^CD8^−^ double negative thymocyte subsets (DN1-4, with different expression of CD44 and CD25 markers). **(H–K)** Total double negative (DN), double positive (DP; CD4^+^CD8^+^), and single positive (SP; CD4^+^ or CD8^+^) thymocytes in control (*n* = 10) and G-ARKO (*n* = 10) mice. ^*^P < 0.05, ^**^P < 0.01, ^***^P < 0.001 (Mann-Whitney *U*-test); all bars indicate means; circles represent individual mice, error bars indicate SEM.

We first studied the effect of general AR depletion on thymopoiesis; mice with a general knockout of the AR (G-ARKO) had increased thymus weight and cellularity compared to littermate (Pgk-Cre^+^) controls (Figures 1A,B). The number of thymocytes was increased at all stages of T lymphopoiesis, including the early double negative (CD4^−^CD8^−^) stages (Figures 1C–H) as well as more mature double positive (CD4^+^CD8^+^) and single positive (CD4^+^ or CD8^+^) cells (Figures 1I–K).

As G-ARKO mice are both AR- and testosterone-deficient (17), we next castrated G-ARKO and control littermate mice and replaced with a physiological dose of testosterone (17), to distinguish the effects of testosterone deficiency from AR deficiency on thymus weight. While testosterone replacement reduced thymus weight in castrated control mice, it did not affect the thymus weight of G-ARKO mice (Supplemental Figure 1), showing that the effect of testosterone on thymus weight is completely AR-dependent.

### Unchanged Thymopoiesis in T-ARKO Mice

We next searched for the target cell for the effects of androgens on thymopoiesis. To assess if androgens/AR affect T cell homeostasis through a T cell-intrinsic mechanism, we generated T cell-specific ARKO (T-ARKO) mice using the pLCK-Cre^+^ construct, and quantified thymocytes in these mice and littermate (pLCK-Cre^+^) controls. Despite a highly efficient knockout of AR exon 2 *g*DNA in CD3^+^ T cells (Supplemental Figure 2A), T-ARKO mice had unchanged thymus weight and cellularity, and the number of T cell precursors were unaffected by AR-deficiency in T cells (Supplemental Figures 2B–G), showing that the enhanced thymopoiesis in AR deficiency is not T cell-intrinsic.

### Increased Thymopoiesis in E-ARKO Mice

**Figure 2:**
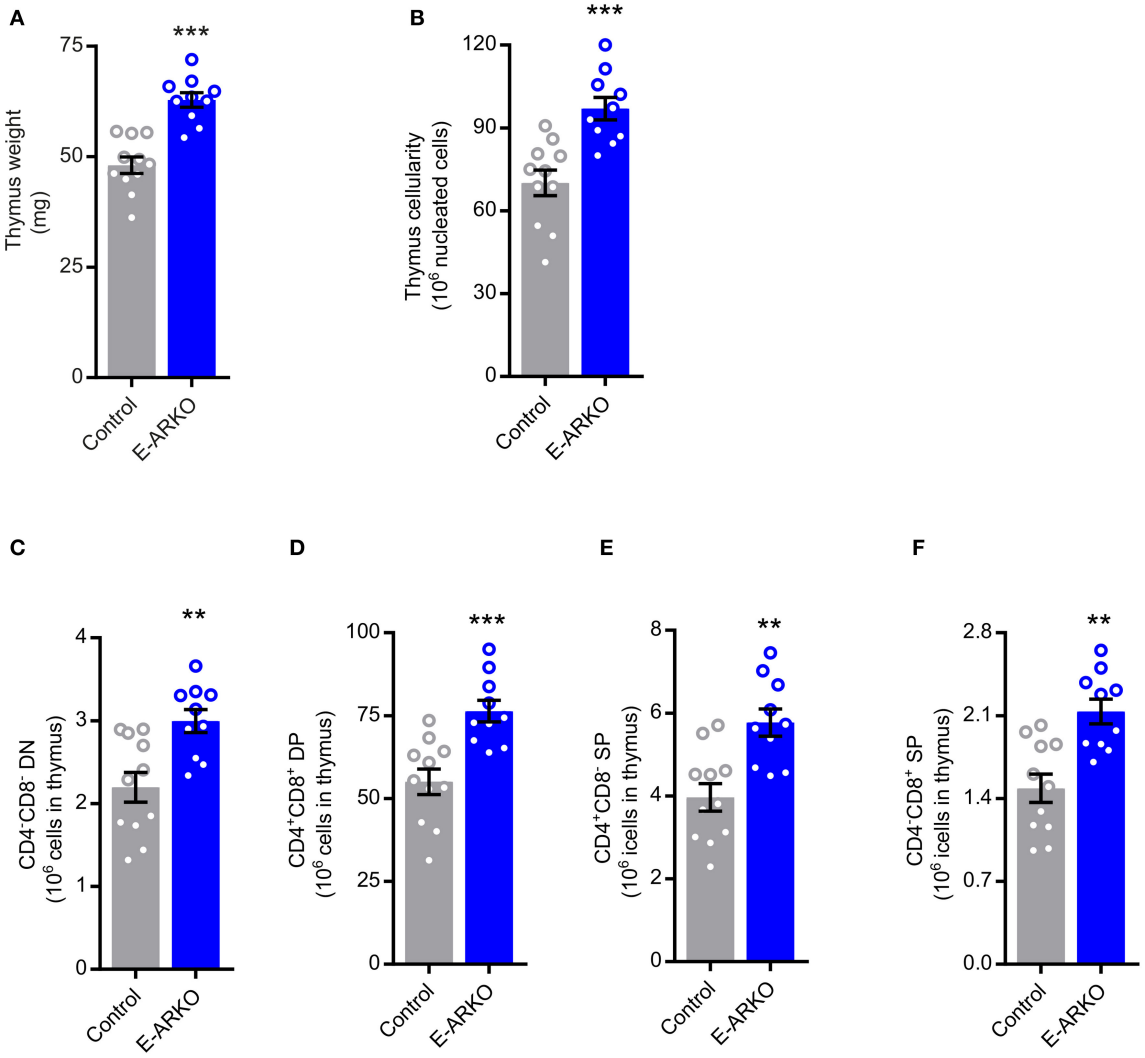
Increased thymopoiesis in mice with depletion of AR in epithelial cells (E-ARKO). **(A,B)** Thymus weight and cellularity in control (K5-Cre^+^; *n* = 11) and E-ARKO (AR^fl^K5-Cre^+^; *n* = 10) male mice. **(C–F)** Number of double negative (DN; CD4^−^CD8^−^), double positive (DP; CD4^+^CD8^+^), and single positive (SP; CD4^+^ or CD8^+^) thymocytes in control (*n* = 11) and E-ARKO (*n* = 10) mice. ^**^P < 0.01, ^***^P < 0.001 (Mann-Whitney *U*-test); all bars indicate means; circles represent individual mice, error bars indicate SEM.

As factors secreted by the thymic stroma are known to influence the thymic microenvironment to support T lymphopoiesis (18) and the AR is expressed in thymic epithelial cells (TECs) (6), we hypothesized that TECs are targets for AR-dependent actions on T cell homeostasis. Therefore, we generated epithelial cell-specific ARKO (E-ARKO) mice using a K5-Cre^+^ construct (19), where Cre is expressed under the control of the K5-promotor in epithelial cells [model described in (20)]. Confirming our hypothesis, E-ARKO mice displayed increased thymus weight and cellularity (Figures 2A,B) compared to littermate (K5-Cre^+^) controls. In line with the results in G-ARKO mice, the number of thymocytes was increased in E-ARKO at all stages of T lymphopoiesis (Figures 2C–F).

### Increased Staining of CCL25 in Thymi of E-ARKO Mice

**Figure 3:**
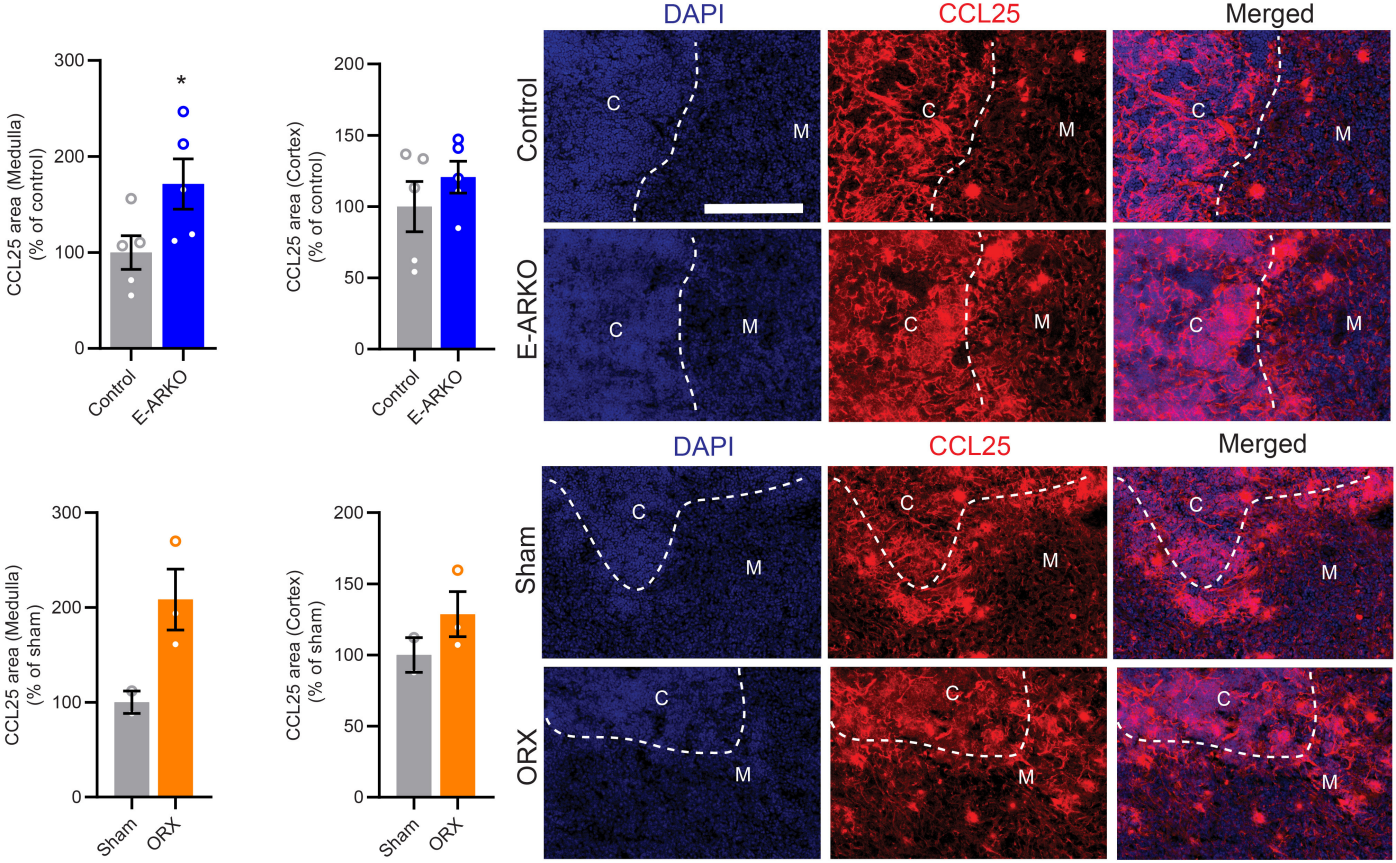
Increased staining of CCL25 in thymi of E-ARKO mice. Quantification of CCL25-stained area in thymic medulla and cortex of E-ARKO and control mice and mice that were sham-operated or castrated (orchiectomized; ORX) at 4 weeks of age; tissues were collected at 34 weeks of age. Thymic sections were stained for CCL25 (red) and nuclei were stained by 4',6-Diamidino-2-Phenylindole (DAPI; blue). Scale bar = 200 μm. ^*^P < 0.05 vs. control (Mann-Whitney *U*-test). Bars indicate means, error bars indicate SEM, and circles represent individual mice.

CCL25 has been shown to be central to the effects of testosterone deficiency on thymopoiesis (9). Therefore, we next studied the E-ARKO effect on the expression of CCL25 using thymic sections. Indeed, compared to control mice, E-ARKO mice showed an increased CCL25-positive area in medulla, but not cortex (Figure 3). A similar pattern was found in castrated (testosterone-deficient) mice (Figure 3).

### TEC Shift in E-ARKO Mice

**Figure 4:**
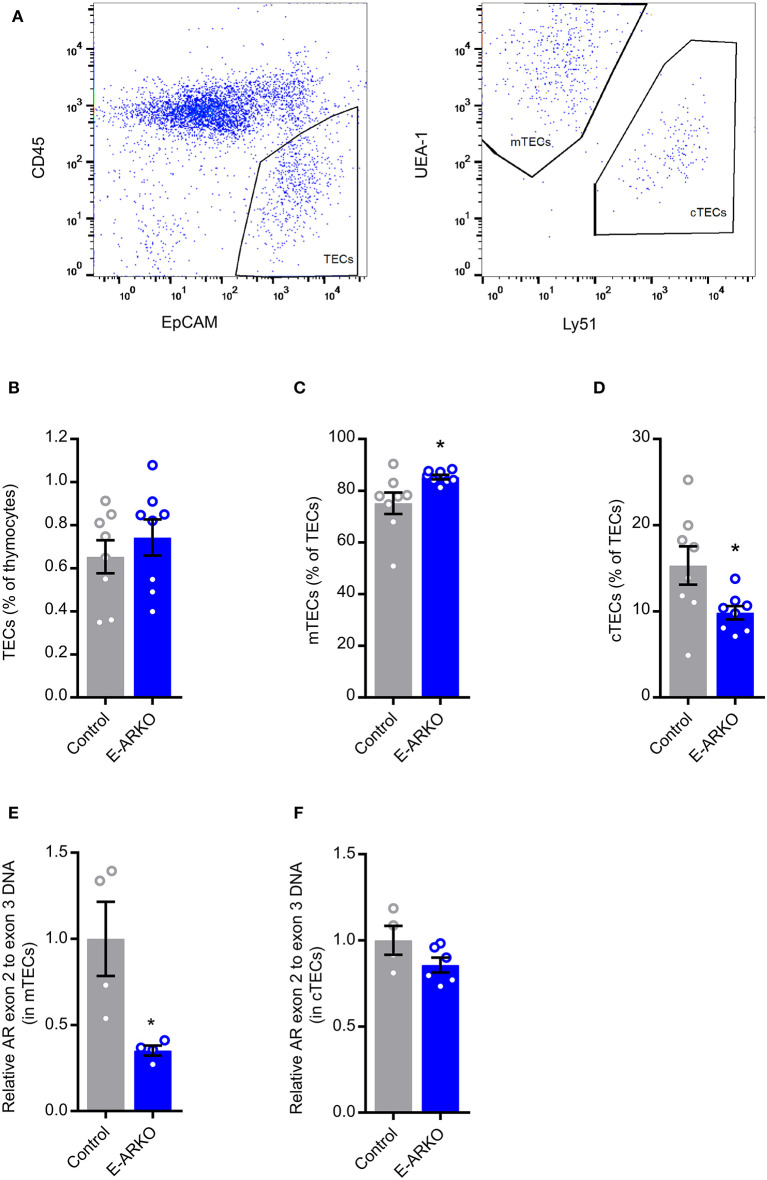
TEC shift in E-ARKO mice. **(A)** Gating strategy for TECs. **(B–D)** Relative numbers of all thymic epithelial cells (TECs), cortical (cTEC; CD45^−^ EpCAM^+^ UEA1^−^ Ly51^+^), and medullary (mTEC; CD45^−^ EpCAM^+^ UEA1^+^ Ly51^−^) TEC in control and E-ARKO male mice; *n* = 8/group. **(E)** Assessment of AR knockout by measurement of exon 2 gDNA in mTECs from 4-week-old control (K5-Cre^+^; *n* = 4) and E-ARKO (AR^fl^K5-Cre^+^; *n* = 4) male mice. **(F)** Assessment of AR knockout by measurement of exon 2 gDNA in cTECs from 8-week-old control (K5-Cre^+^; *n* = 4 pools, 2 mice/pool) and E-ARKO (AR^fl^K5-Cre^+^; *n* = 6 pools, 2 mice/pool) male mice. ^*^P < 0.05 (Mann-Whitney *U* test); all bars indicate means; circles represent individual mice, error bars indicate SEM.

Recent data suggest that castration of male mice results in relatively reduced cTEC and increased mTEC number (15). Quantifying mTEC (CD45^−^ EpCAM^+^ UEA1^+^ Ly51^−^) and cTEC (CD45^−^ EpCAM^+^ UEA1^−^ Ly51^+^) populations (Figure 4A), we saw a similar pattern in E-ARKO, i.e., a relative reduction of cTECs and a minor increase in the relative number of mTECs. By contrast, the overall TEC fraction in thymus was not different between E-ARKO and controls (Figures 4B–D). Analyzing the knockout of AR exon 2 gDNA in mTECs and cTECs, there was a partial (mean 65%) depletion of AR exon 2 in mTECs, but no significant depletion in cTECs (Figures 4E,F).

### Increased Peripheral T cells and Recent Thymic Emigrants (RTEs) in E-ARKO Mice

**Figure 5:**
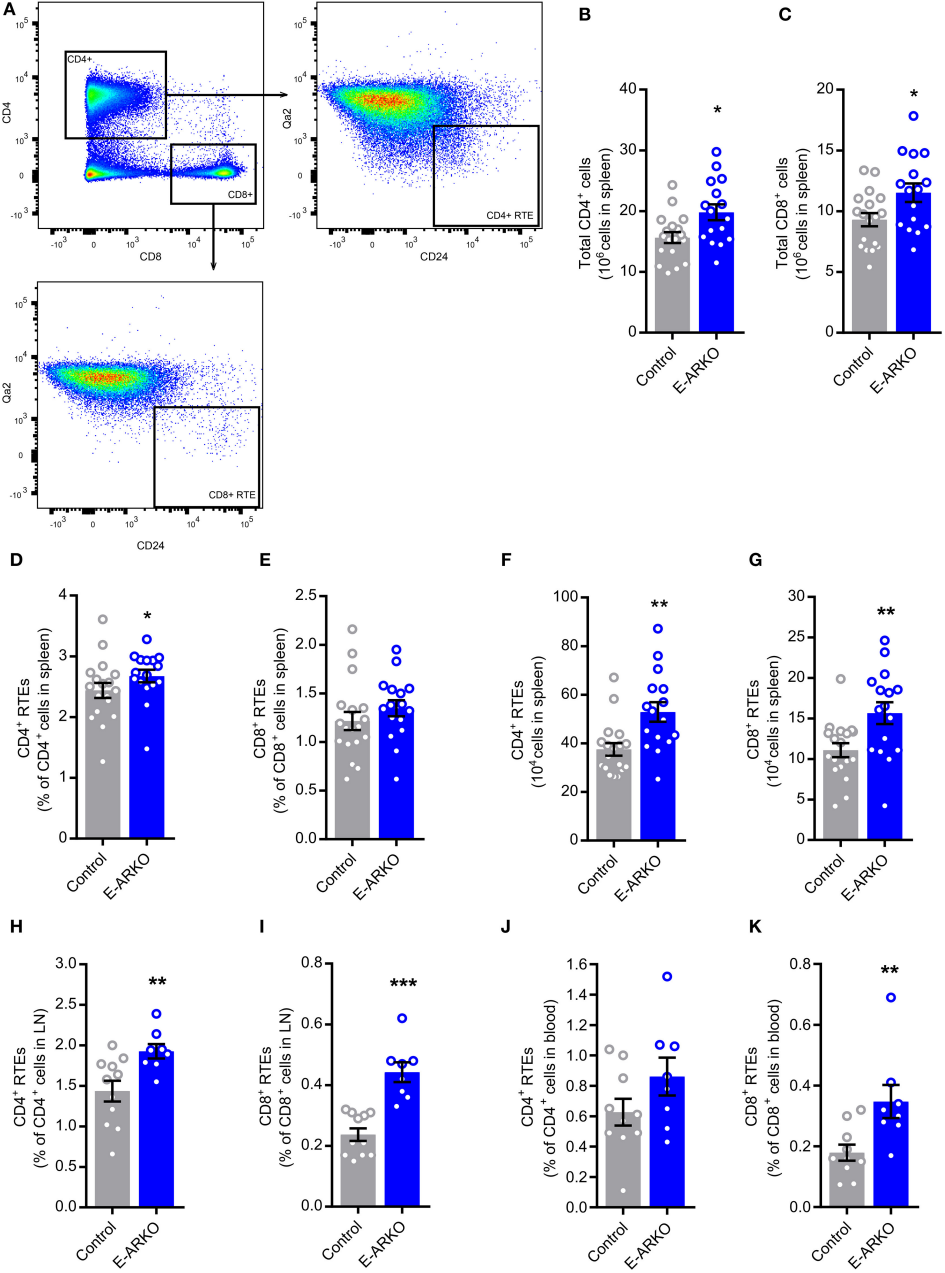
Increased numbers of peripheral T cells and recent thymic emigrants (RTEs) in E-ARKO mice. **(A)** Gating strategy for CD4^+^ and CD8^+^ T cells and CD4^+^ and CD8^+^ RTEs. **(B,C)** Total numbers of CD4^+^ and CD8^+^ T cells in spleen from control (*n* = 18) and E-ARKO (*n* = 15) male mice. **(D–G)** Relative and total number of CD4^+^ and CD8^+^ RTEs (Qa2^lo^CD24^hi^ CD4^+^ or Qa2^lo^CD24^hi^ CD8^+^) in spleen from control (*n* = 18) and E-ARKO (*n* = 15) mice. **(H,I)** Relative number of CD4^+^ and CD8^+^ RTEs in lymph nodes (LN; pooled inguinal and para-aortic lymph nodes) from control (*n* = 11) and E-ARKO (*n* = 8) mice. **(J,K)** Relative number of CD4^+^ and CD8^+^ RTEs in blood from control (*n* = 10) and E-ARKO (*n* = 8) mice. ^*^P < 0.05, ^**^P < 0.01, ^***^P < 0.001 (Mann-Whitney *U*-test); all bars indicate means; circles represent individual mice, error bars indicate SEM.

We next studied the peripheral T cell pool and the frequency of RTEs in secondary lymphoid organs of E-ARKO mice. We defined RTEs as Qa2^lo^CD24^hi^ CD4^+^ or Qa2^lo^CD24^hi^ CD8^+^ cells (13) (Figure 5A). E-ARKO showed an increased number of both CD4^+^ and CD8^+^ T cells in spleen (Figures 5B,C). In E-ARKO mice, RTEs constituted a significantly greater part of total CD4^+^ (+10%, *P* < 0.05), with a similar trend for CD8^+^ (+15%, *P* = 0.13) T cells in spleen (Figures 5D,E). The total number of both CD4^+^ and CD8^+^ RTEs were increased in spleen of E-ARKO mice (Figures 5F,G).

In pooled inguinal and para-aortic lymph nodes, the frequency of both CD4^+^ (+33%, *P* < 0.01) and CD8^+^ RTEs (+61%, *P* < 0.001) were increased in E-ARKO compared to control mice (Figures 5H,I). In blood, the frequency of CD8^+^ RTEs (+94%, *P* < 0.01), but not CD4^+^ RTEs (+37%, *P* = 0.15) were significantly increased (Figures 5J,K). Taken together, peripheral RTEs were increased in E-ARKO mice, with some variation in effect between compartments.

## Discussion

In this study, we have utilized cell-specific AR knockout mouse models to investigate how androgens/AR affects T cell homeostasis. We show that epithelial cells are a target for androgen/AR-mediated actions on thymopoiesis, splenic T cells, and RTEs in secondary lymphoid organs.

Here we identified the TEC as a target cell for androgen regulation of thymus size, in accordance with data of increased thymopoiesis in both G- and E-ARKO models (14). Our data are also in accordance with data demonstrating a hematopoietic cell-extrinsic, rather than -intrinsic, AR-dependent inhibition of thymopoiesis (6). In line with the previous data (14), we found that all thymic T lymphocyte stages, from double negative through single positive cells, were increased in both G- and E-ARKO mice.

Notably, the effect size differed between the two models; G-ARKO had a doubling of thymus cellularity, whereas thymus cellularity was increased by ~40% in E-ARKO. This divergent effect size might result from an incomplete knockout of the AR in TECs of E-ARKO mice as shown here. Further, recent data support that complete androgen blockade affects thymus cellularity partly by increasing the number of thymus-seeding precursors from the bone marrow (21). This bone marrow effect of androgens/AR may also contribute to the discrepancy between the two models, as G-ARKO mice are both AR- and testosterone-deficient due to underdeveloped testes in this model, while E-ARKO mice have normal testicular development and unaltered levels of testosterone (17, 20). In an effort to distinguish the effects of testosterone- vs. AR-deficiency in G-ARKO mice, we castrated the mice to remove endogenous testosterone production and supplemented the mice with a physiological dose of testosterone (17). Our results show that the effect of testosterone on thymus size is completely AR-dependent.

In the present study, we found that the RTEs in secondary lymphoid organs (spleen and lymph nodes) were increased in E-ARKO mice, which has not been reported previously. Overall, these data suggest that TECs are androgen target cells for the regulation of both thymus size and export of RTEs. Indeed, thymus size is an important determinant of thymic output of RTEs, independently of other factors such as age (22). Our data are in line with previous studies suggesting that the fraction of RTEs increases in the periphery after castration and/or androgen deprivation therapy of both mice and humans (3, 5). As RTEs are precursors to mature T cells, the increased frequency of RTEs is a plausible explanation for the increased number of splenic T cells in E-ARKO mice, which also were found by Lai and coworkers (14). Further, E-ARKO mice showed enhanced donor-derived thymocyte and splenocyte numbers after bone marrow transplantation (14).

Expressed as the percentage out of the total pool of T cells, we found that the frequency of CD4^+^ RTEs (among CD4^+^ T cells) was increased in spleen of E-ARKO mice, with a similar trend for CD8^+^ RTEs (among CD8^+^ T cells). In the lymph nodes and blood, there seems to be a slightly larger E-ARKO effect on CD8^+^ RTEs as compared to CD4^+^ RTEs. Further, there may be a slightly more prominent effect in the lymph nodes as compared to the spleen for both CD4^+^ and CD8^+^ RTEs. Although the latter trends require confirmation, they raise the question whether AR depletion in TECs alters the peripheral trafficking of RTEs by shaping RTE properties. To our knowledge, no studies have yet addressed possible androgen/AR-mediated regulation of the homing patterns of RTEs or regulation of their expression of adhesion molecules, chemokines, and/or chemokine receptors known to be involved in the migration of RTEs to secondary lymphoid organs (13).

We used K5-driven Cre recombinase expression to target the AR in TECs. Few studies have reported the degree of DNA depletion in TECs using the K5-Cre construct, as the Foxn1-Cre system has been most widely used for TEC-specific targeting (16). However, using K5-Cre, Lai et al. detected Cre reporter signal in both cortex and medulla (14). In the present study, we found a mean knockout degree of around 65 % in mTECs of E-ARKO mice, while we could not demonstrate a deletion of AR in cTECs. In addition to most differentiated mTECs, common thymic epithelial stem/progenitor cells express K5 (23), but it is possible that K5 expression in early TECs is not general and/or sufficient to drive Cre expression that results in depletion across TEC subsets. Notably, a recent study reported that castration results in reduced cTEC numbers, which the authors coupled to increased apoptosis of cTECs and increased differentiation of cTEC-phenotype progenitors into mTECs (15). Theoretically, if AR depletion in TECs affects cTEC survival and/or differentiation, quantification of AR knockout degree in cTECs may be biased by preferential loss of AR-depleted cTECs/cTEC-phenotype progenitors through these processes. As previously reported after castration (15), we found a shift in the relative number of mTECs and cTECs in E-ARKO mice; thus, the effect of androgen deficiency on mTEC/cTEC ratio is partially mimicked by AR depletion in TECs using K5-driven Cre expression. However, our result contrasts that of Lai et al. who found proportionally increased cellularity of mTEC and cTEC fractions in E-ARKO mice, although they used slightly different markers for defining cTECs (14).

Our finding of no detectable AR deletion in cTECs of E-ARKO mice is unexpected given the thymopoiesis phenotype of these mice, as cTEC functions are known to be important for T cell expansion and development (16). However, molecular delineation of individual TEC subpopulations is a work in progress (16), and new data emphasizes the role of medullary stromal signals for the function of the cortical stroma (24), suggesting an important interplay between different TEC fractions. Notably, we found increased staining of CCL25 in the medulla, but not cortex, of E-ARKO mice, similar to the pattern of castrated (testosterone-deficient) mice. CCL25 has been shown to be an important mediator of the effects of testosterone deficiency on thymopoiesis through increased uptake of early T-lineage progenitors and regulation of the maturation of double negative thymocytes (9). Thus, these mechanisms may possibly be mimicked in the E-ARKO model. Our findings indicate that the androgen/AR-mediated regulation of CCL25 mainly occurs in the medulla, which is in accordance with previous data showing that the greatest increase in CCL25 production after castration is by UEA^+^ mTECs (9).

Patients with androgen deficiency, such as Klinfelter patients, have both increased number of T cells (4) and increased risk of autoimmune diseases (25). However, whether androgen-mediated modulation of thymus/TEC biology plays a role in T cell-dependent disorders remains largely unclear. Our group recently showed that E-ARKO mice display increased atherosclerosis, which was abolished by prepubertal thymectomy (20). Although the mechanism underlying increased thymus-dependent atherosclerosis in E-ARKO mice remains to be established, it may theoretically relate to various TEC functions such as negative selection, regulatory T cell formation, or RTE formation (26). To date, it remains unclear whether the E-ARKO mice are prone to other inflammatory and/or autoimmune disorders. Deciphering whether the thymic epithelium is a target compartment for androgen/AR-mediated regulation of inflammation and autoimmunity, and defining the mechanisms mediating such effects, will be important tasks for future studies.

In conclusion, we demonstrate that the thymic epithelium is a target compartment for androgen/AR-mediated regulation of thymopoiesis and consequently the generation of RTEs.

## Methods

### Animals

G-ARKO and E-ARKO male mice were generated as previously described (17, 20). T-ARKO and E-ARKO male mice were generated by breeding AR^+/flox^ female mice with male pLCK-Cre^+^ mice (Stock no. 003802, B6.Cg-Tg(LCK-Cre)548Jxm/J, Jackson laboratory, Bar Harbor, Maine, USA), and K5-Cre^+^ mice (19), respectively. Because our initial assessments of androgen status (wet weight of androgen sensitive organs) and thymus weight and cellularity revealed no differences between AR^+^ and AR^flox^ males, Cre^+^ littermates without the AR^flox^ construct were used as controls for subsequent experiments. In all experiments the different ARKO mice were compared to littermate controls and the mice were on a C57BL/6 ApoE constitutive knockout background (B6.129P2-Apoetm1UncN11, Taconic). We assessed AR, Cre, and Zfy (for gender) by PCR amplification of genomic DNA (gDNA) (27). The mice were housed in a temperature- and humidity-controlled room with a 06:00–18:00 h light cycle and consumed a soy-free diet (R70, Lantmännen) and tap water *ad libitum*. All animal studies were conducted in compliance with local guidelines and The Ethics Committee on Animal Care and Use in Gothenburg approved all procedures.

### Castration and Testosterone Replacement

In a separate experiment, G-ARKO mice and littermate controls were bilaterally orchiectomized and implanted subcutaneously with a small slow-releasing pellet containing placebo or a physiological dose of testosterone (25 μg/day; Innovative Research of America, Sarasota, FL, USA) as previously described (17).

### Tissue Collection

At 10–16 weeks of age (unless otherwise stated), the mice were anesthetized and blood was drawn from the left ventricle and collected in EDTA tubes (Microvette, Sarstedts). The mice were perfused with saline under physiological pressure and tissues (thymus, spleen, inguinal lymph nodes, and para-aortic lymph nodes) were dissected and kept in PBS on ice.

### Cell Preparation and Flow Cytometry Analysis of T lymphocytes

Single cells from thymus, spleen, and lymph nodes were prepared by passing the tissue through a 40 μm cell strainer (Thermo Fisher) using PBS and a syringe plunger. Erythrocytes in blood and spleen were lyzed in lysis buffer (0.16 M NH_4_Cl, 0.13 M EDTA, and 12 mM NaHCO_3_), the cells were washed in flow cytometry buffer (2% fetal bovine serum and 2 mM EDTA in PBS) and counted in an automated cell counter (Sysmex). After FcR-blockage (anti-mouse CD16/CD32, BD Biosciences), antibodies specific for the following markers were used: CD4 (GK1.5, Biolegend or RM4-5, BD Biosciences), CD8a (53-6.7, Biolegend), CD44 (IM7, Biolegend), CD25 (PC61, BD Biosciences), CD24 (M1/69, BD Biosciences), and Qa-2 (1-1-2, BD Biosciences). Immunostained cells were analyzed on a FACS Canto II, Accuri C6, or FACS Aria (BD Biosciences). Data were analyzed using FlowJo (Tree Star) and fluorochrome-minus-one staining was used as controls.

### Thymus Sections

Cryosections (10 μm) of thymus were air dried for 2 hrs and stored at −20°C. Sections were fixed for 5 min in 2% formaldehyde, permeabilized with 0.1% Triton-X for 4 min, blocked in 1% bovine serum albumin and Fc-receptor blocking antibody (anti-mouse CD16/32, clone 2.4G2; BD Biosciences; 1:100) for 30 min at r.t. and incubated with a primary polyclonal goat anti CCL25 antibody (InVitrogen; cat no. PA5-47662; 1:500) in blocking buffer overnight at 4°C. Sections were then washed 3 × 5 min in PBS, incubated with secondary antibody F(ab)2 AF594-conjugated donkey anti-goat IgG (Jackson ImmunoResearch; 1:300) in blocking buffer for 1.5 hrs at r.t, stained in 1 μg/mL DAPI in PBS for 3 min, washed as above and mounted with ProlongGold mounting medium (Life Technologies). The thymic medulla and cortex areas were manually delineated by a blinded observer and the CCL25 positive area was quantified using Visiopharm Integrator System (version 2017.2).

### Cell Preparation and Flow Cytometry Sorting of Thymic Epithelial Cells (TECs)

The thymi were fragmented and excess of thymocytes washed away by mechanical disruption. TECs were released by enzymatic digestion. Briefly, the thymic fragments were incubated in digestion medium (0.5 U/mL Liberase TM (Roche), 0.2 mg/mL DNase I (Roche) in DMEM/F12) at 37°C with gentle mixing for 20 min. The released cells were transferred into cold flow cytometry buffer. New pre-warmed digestion medium was added to remaining thymic fragments for two more consecutive incubations, to completely dissolve the tissue. The released cell fractions were filtered through a 100 μm cell strainer (BD Biosciences), washed and counted. Cells from the two latter fractions were pooled for analysis. After incubation with FcR block (CD16/CD32, BD Biosciences), antibodies against CD45 (30-F11, BD Biosciences) and EpCAM/CD326 (G8.8, BD Biosciences), Ly51 (6C3, BD Biosciences) and the biotinylated lectin UEA-1 (Vector Laboratories) were added. The cells were washed, resuspended in flow cytometry buffer, and filtered through a 100 μm cell strainer. mTECs (CD45^−^ EpCAM^+^ UEA1^+^ Ly51^−^) and cTECs (CD45^−^ EpCAM^+^ UEA1^−^ Ly51^+^) were sorted on a SY3200 cell sorter (SONY Biotechnology Inc.).

### AR DNA Quantification

In the ARKO mouse model exon 2 of the AR gene is excised (27) and the presence of exon 2 versus exon 3 was used to quantify the efficacy of the AR knockout. CD3^+^ cells were isolated from thymus using positive selection with MACS (magnetic-activated cell sorting) CD3 microbeads (Miltenyi Biotec). Genomic DNA from CD3^+^ cells was isolated using DNeasy blood and tissue kit (Qiagen) according to the manufacturer's instructions. Genomic DNA amplification was detected using SyBR green master mix (Applied Biosystems) in an ABI Prism 7900HT Sequence Detection System (Applied Biosystems). The following primer pairs were used: AR exon 2, forward GGACCATGTTTTACCCATCG and reverse CCACAAGTGAGAGCTCCGTA; and AR exon 3, forward TCTATGTGCCAGCAGAAACG and reverse CCCAGAGTCATCCCTGCTT. Ct values for AR exon 2 were normalized to Ct values for AR exon 3 using the 2^−ΔΔct^ method (28).

### Statistics

Statistical evaluations were performed with Prism software (GraphPad Software, Inc.). The non-parametrical Mann-Whitney *U*-test was used for all for two-group comparisons and Kruskal-Wallis followed by Mann-Whitney U for comparisons between four groups. *P*-values of < 0.05 were considered statistically significant. Unless otherwise specified, results are represented as mean ± SEM.

## Data Availability Statement

The datasets generated for this study are available on request to the corresponding author.

## Ethics Statement

The animal study was reviewed and approved by The Ethics Committee on Animal Care and Use in Gothenburg.

## Author Contributions

AW, ML, IJ, ES, AS, SL, JF, PF, HC, OE, and ÅT designed the studies. AW, ML, IJ, ES, AS, and JF conducted experiments and/or acquired data. AW, ML, IJ, AS, SL, and ÅT analyzed the data. AW, ML, IJ, and ÅT wrote the manuscript. AW, ML, IJ, ES, AS, SL, JF, PF, HC, OE, and ÅT revised the manuscript. All authors contributed to the article and approved the submitted version.

## Conflict of Interest

The authors declare that the research was conducted in the absence of any commercial or financial relationships that could be construed as a potential conflict of interest.
