# Supplementary material for: Androgen Receptors in Epithelial Cells Regulate Thymopoiesis and Recent Thymic Emigrants in Male Mice
Source: Front Immunol. 2020 Jun 29;11:1342. doi: 10.3389/fimmu.2020.01342 (PMC7344216; doi:10.3389/fimmu.2020.01342)
Supplement: Supplementary file 1 [file Data_Sheet_1.PDF]

SUPPLEMENTARY FIGURES

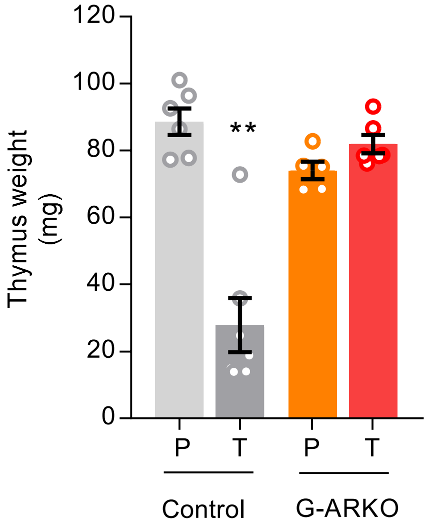

Figure S1. Thymus weight in castrated control and G-ARKO mice with and without testosterone replacement. Four weeks before tissue collection, 8-week-old general androgen receptor knockout (G-ARKO; AR^{fl}Pgk-Cre^{+}) mice and littermate controls (Pgk-Cre^{+}) were bilaterally orchiectomized (ORX) and implanted subcutaneously with a small slow-releasing pellet containing placebo (P) or a physiological dose of testosterone (T). **$p<0.01$ (Kruskal-Wallis followed by Mann-Whitney U test); all bars indicate means; circles represent individual mice, error bars indicate SEM.

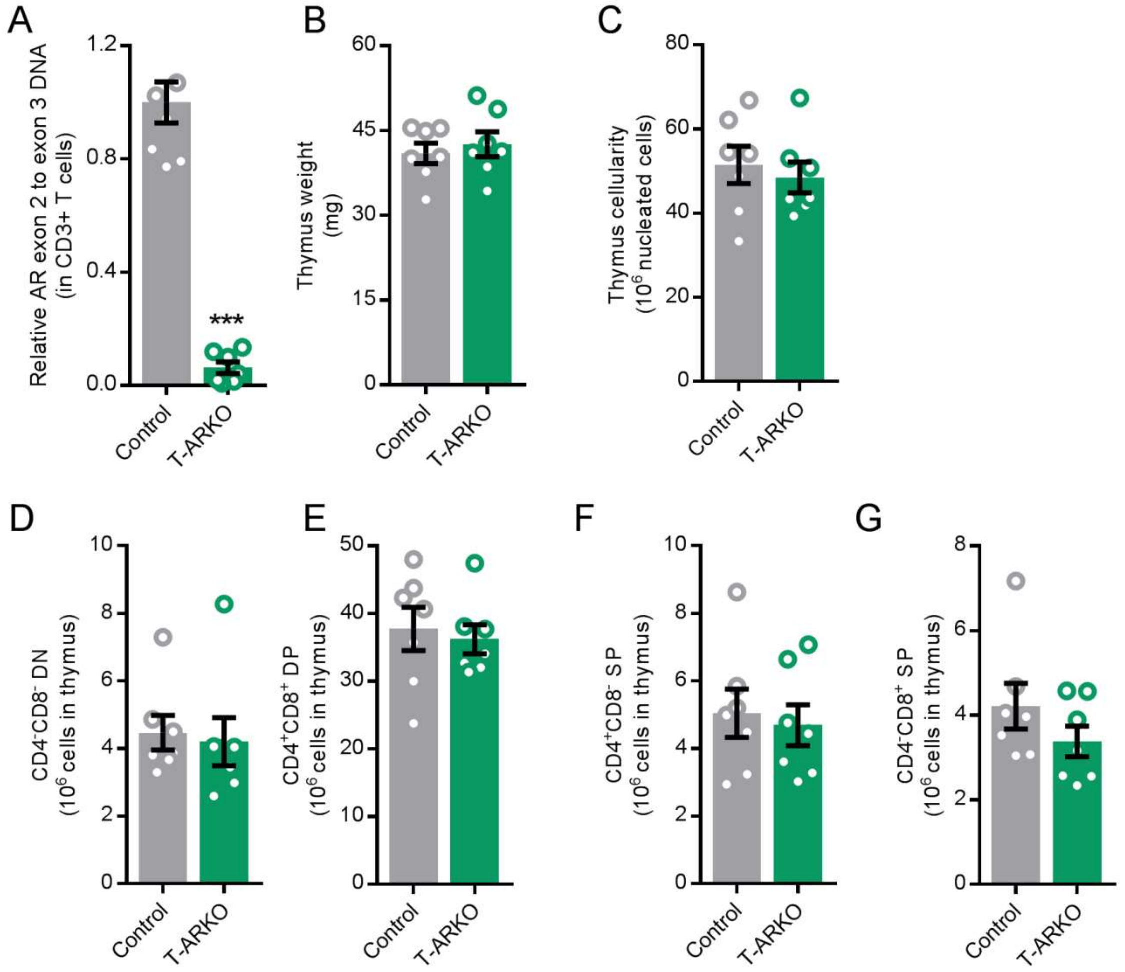

Figure S2. T cell-specific ARKO (T-ARKO) mice. (A) Assessment of AR knockout by measurement of exon2 *g*DNA in thymic T-cells from control (pLCK-Cre$^+$; n=8) and T-ARKO (AR^{fl}pLCK-Cre$^+$; n=7) male mice. (B-C) Thymus weight and cellularity in control (n=7) and T-ARKO (n=8) male mice. (D-G) Number of double negative (DN; CD4$^-$CD8$^-$), double positive (DP; CD4$^+$CD8$^+$), and single positive (SP; CD4$^+$ or CD8$^+$) thymocytes in control (n=7) and T-ARKO (n=7) mice. ***P <0.001 (Mann-Whitney U test); all bars indicate means; circles represent individual mice, error bars indicate SEM.
